# Serum biomarkers as personalised medicine for diagnostic and therapeutic approaches of hepatocellular carcinoma

**DOI:** 10.1186/s43046-026-00342-1

**Published:** 2026-03-09

**Authors:** Muhammad Taher, Muhamad Aqil Ikram Muhamad Asri, Nur Damia Abdul Rahman, Nur Eleisha Zamzuri, Nur Syazwani Mohammad Farizal, Junaidi Khotib, Deny Susanti, Nurul Athirah Hasdan, Muhammad Salahuddin Haris, Rita Rakhmawati

**Affiliations:** 1 Kulliyyah of Pharmacy, International Islamic University Malaysia, Kuantan, Malaysia; 2 Department of Chemistry, Kulliyyah of Science, International Islamic University Malaysia, Kuantan, Pahang Malaysia; 3 Faculty of Pharmacy, Airlangga University, Surabaya, Indonesia; 4 Department of Pharmacy, Sebelas Maret University, Surakarta, Indonesia; 5 Department of Pharmacy, Faculty of Pharmacy and Health Sciences, Royal College of Medicine Perak, Universiti Kuala Lumpur, Ipoh, Perak Malaysia

**Keywords:** Hepatocellular carcinoma, Serum biomarkers, Diagnostic, Therapeutic, Alpha fetoprotein, Des-γ- carboxyprothrombin, Glypican-3

## Abstract

Hepatocellular carcinoma (HCC) is a critical public health concern due to its rising incidence and mortality. Early diagnosis is challenging due to nonspecific symptoms and limitations of traditional methods. This study explores the potential of three key biomarkers, serum alpha-fetoprotein (AFP), des-γ-carboxyprothrombin (DCP), and glypican-3 (GPC3), in advancing personalized medicine for HCC, focusing on their roles in diagnostics and therapeutics. We conducted a literature review using specific keywords to narrow our search. Our findings indicate that AFP and DCP are primarily used for diagnostics, while GPC3 serves both diagnostic and therapeutic purposes. AFP is commonly used in late-stage detection due to its limited sensitivity and specificity in early stages, which can lead to false diagnoses. DCP plays a significant role as both a diagnostic tool and therapeutic target, while GPC3 is used to differentiate malignant from non-cancerous liver tissues. The review highlights the mechanisms, roles, and personalised treatments associated with these biomarkers, while also addressing challenges such as biopsy issues and funding limitations. In conclusion, the development and improvement of AFP, DCP, and GPC3 as biomarkers for the diagnostics and treatment of HCC are important. Thus, their limitations should be addressed as these biomarkers play a crucial role in modern cancer therapy and are critical for increasing the survival rates of HCC patients.

## Introduction

The steadily increasing incidence and mortality rates of liver cancer worldwide continue to position it as a critical public health concern. As the predominant type of liver cancer, hepatocellular carcinoma (HCC) accounted for 906,000 new cases and 830,000 deaths in 2020 alone, thus underscoring its status as the third leading cause of cancer mortality and the sixth most diagnosed malignancy worldwide [1]. Unlike many other human malignancies, the risk factors for HCC are well documented. In most cases, HCC is commonly associated with advanced hepatic fibrosis or cirrhosis that arises from chronic liver disease. This is particularly apparent in cases of liver damage attributed to hepatitis B virus (HBV) or hepatitis C virus (HCV) infections, as well as excessive alcohol consumption [2]. This correlation is further supported by [3], who reported that approximately 80% of HCC cases occur in sub-Saharan Africa and Eastern Asia, regions with a high prevalence of chronic HBV carriers. This highlights the critical need for improved early detection strategies to control HCC better. However, due to the lack of clear symptoms, early diagnosis remains challenging, often resulting in detection at advanced stages where treatment options are limited and the prognosis is poor [4]. Techniques like imaging and tissue biopsy have been crucial in diagnosing various cancers, but they often fall short in sensitivity and specificity when it comes to detecting cancer in its early stages. These methods are limited by their inability to identify subtle molecular changes associated with early-stage disease and their potential for yielding false positives or negatives [5].

To address this challenge, one of the key concepts is personalised medicine, which focuses on tailoring treatment to specific individuals rather than using a one-size-fits-all method [6]. While the ‘5 Rights of Medication Administration,’ which ensures the right patient receives the right medication, dose, time, and route, has provided a foundational framework for safe medication practice, personalised medicine enhances these principles by incorporating individual patient characteristics including genetic and molecular profiles. In this context, biomarkers are invaluable tools. Tumor biomarkers, which are typically proteins detected in blood, urine, or other body fluids, are either produced by the tumor itself or in response to the presence of cancer. They are objectively defined, quantifiable signs that can be used to indicate a biological process, a disease’s stage, or an organism’s reaction to a particular therapeutic intervention [7]. Thereby, they are indispensable as they help avoid broad, empirical treatment approaches resulting in significant savings of both time and resources [8].

In recent years, the method of non-invasive diagnostics utilising biomarkers has gained recognition as an alternative to conventional procedures. The development of advances in research and technology has resulted in the identification and application of several novel HCC biomarkers. Among them are the three most promising biomarkers which are alpha-fetoprotein (AFP), des- γ - carboxyprothrombin (DCP) and glypican-3 (GPC3) In this paper, we delve into the potential of these three biomarkers in advancing personalised medicine for HCC while highlighting their role in improving diagnostic accuracy and facilitating tailored therapeutic strategies.

## Methodology

A thorough literature review was conducted, emphasising serum biomarkers used for diagnostic and therapeutic approach for the treatment of hepatocellular carcinoma. Original and clinical studies were primarily published in English and non-systematically obtained from various databases, including Scopus, Google Scholar, ScienceDirect, ResearchGate, ProQuest Health & Medical Complete, PubMed, and the National Library of Medicine. The search strategy employed targeted keywords such as “Hepatocellular carcinoma”, “HCC”, “GPC3”, “cancer”, “Glypican-3”, “Des-γ- carboxy prothrombin”, “Alpha-fetoprotein”, “AFP”, “DCP”, “DCP-Met-JAK1-STAT3”, “angiogenesis”, “Personalized medicine”, “Tumour biomarkers”, “limitations”,”Serum biomarkers”, “diagnostics”, “mechanism”, “CAR-M”, “vaccine” to effectively find and filter relevant articles on Scopus, Mendeley and other article-based search engines.

## Alpha-fetoprotein (AFP)

AFP is one of the serum biomarkers that is widely used in diagnosis of hepatocellular carcinoma. According to [9] additional risk factors of HCC include male sex, advanced age (over 65 years), the presence of liver cirrhosis, chronic alcohol consumption, diabetes mellitus, metabolic syndrome, hypoalbuminemia, thrombocytopenia and elevated levels of AFP. An AFP tumor marker test is a laboratory blood test designed to quantify the concentration of AFP in a blood sample. However, the sensitivity for detecting the early stages of HCC progression is limited. This is very challenging because it can lead to false-positive results associated with other liver-related conditions such as cirrhosis and acute hepatitis [10]. Serum AFP levels are elevated in individuals with HCC as well as those with benign chronic liver disease, with significant overlap observed between these groups. Consequently, relying solely on serum AFP measurements for monitoring is considered ineffective.

### AFP in HCC diagnosis

Elevation of AFP in the blood indicates that the patients have hepatocellular carcinoma. However, differential diagnosis still presents since the accuracy of AFP in detecting the underlying causes is still low. This is because the serum AFP is not specific to the hepatocytes only, it can be any other related-problems. Baig et al. reported that 30% of HCC patients remained AFB-negative (< 20ng/mL) [11]. The usage of AFB in detecting hepatocellular carcinoma is very beneficial because regular surveillance for early detection is essential to enhance patient outcomes, particularly among high-risk populations [12]. High-risk individuals include hepatitis B, hepatitis C and liver cirrhosis patients are very concerned since untreated liver-related diseases can progress to liver cancer which is aggressive and irreversible. Hence, early detection plays a biggest role in preventing the progression of HCC.

### AFP in cirrhosis with vs. without HCC

Based on the study that had been conducted by [13] the baseline serum alpha-fetoprotein concentrations were within 30 to 460 ng per milliliter (ng/ml) where the median value is 72 ng/ml in the cohort of 32 patients with cirrhosis who were monitored for at least two years without developing HCC. During follow-up, these values fluctuated after two years in the range of 5 to 270 ng/ml (median: 32 ng/ml). In contrast, the baseline serum AFP concentrations were between 30 and 1240 ng/ml (median: 75 ng/ml) increase to the range of 30 to 7080 ng/ml (median: 275 ng/ml) among 33 patients with cirrhosis who developed HCC during follow-up. Notably, only 8 patients in this group exhibited serum AFP concentrations exceeding 500 ng/ml at the time of diagnosis.

From the information that has been collected in the study, there is a significant difference in serum AFP concentration between patients with liver cirrhosis without HCC and patients with liver cirrhosis developed HCC. The higher value of serum AFP concentrations can be seen in patients who develop HCC through liver cirrhosis compared to patients who only have liver cirrhosis. Wei et al. [14] reported that serum AFP level is negative in 20% HCC patients.

### AFP in other cancers

Although AFP was initially regarded as a valuable tumour marker for screening and monitoring patients with hepatocellular carcinoma and yolk sac tumours, it is recognised that certain other malignancies such as colorectal and lung cancers also can synthesise AFP [15]. This is because the expression of primitive phenotypic biomarkers such as GPC3 and Spalt-like transcription factor 4 has been linked to the positivity of AFP. The higher elevation of AFP is not necessary due to HCC but it can also be associated with other types of cancer. Moreover, elevated serum AFP levels can also be observed in individuals with non-cancerous liver conditions such as hepatitis or cirrhosis [16]. On top of that, liver metastasis has been found more frequently in patients with higher preoperative alpha-fetoprotein levels. This is because patients with gastric cancer who exhibit elevated preoperative serum AFP levels are associated with a poor prognosis and an increased likelihood of liver metastasis. Hence, AFP marker is helpful in detecting liver metastasis relating to the re-expression of fetal-like traits like increased proliferation and altered differentiation.

### Combined serum biomarkers for HCC diagnosis

Since there is limitation in the accuracy of HCC diagnosis especially at the early-stage or the level of AFP serum within the normal range, the combination of serum biomarkers in the diagnostic approach of HCC is very recommended in improving accuracy and sensitivity of the results. There are various biomarkers that have been used together with AFP like beta-human chorionic gonadotropin or protein induced by vitamin K absence or antagonist-II (PIVKA-II). Huang et al. [16] mentioned that the combined use of AFP and PIVKA-II enhances sensitivity to 87% across all stages of hepatocellular carcinoma. Specifically, in early-stage HCC, this combination increases sensitivity to 83%. Besides, the combination AFP and PIVKA-II has a role in evaluating and monitoring response to the treatments. The achievement of therapeutic effects by various treatments can be assessed by using these two biomarkers since persistent elevation of either marker post-treatment indicates recurrence disease.

The addition of PIVKA-II in the diagnostic approach is applicable when the symptoms of HCC persist but the AFP serum level is normal. The biomarker PIVKA-II has demonstrated utility in the diagnosis of hepatocellular carcinoma, predicting the recurrence of the disease and assessing high-risk patients when used in conjunction with another biomarker [17]. Hence, AFP and PIVKA-II serve as complementary biomarkers in the management of HCC. The diagnostic limitations by the usage of AFP biomarkers are mitigated by PIVKA-II. This proves that the identification of AFP-negative cases is valuable by the usage of PIVKA-II in combination to enhance accuracy of early-detection, prognostic evaluation and HCC monitoring response.

## Des- γ-carboxy prothrombin (DCP)

Biomarkers possess a crucial role in the management of the HCC by assisting in the diagnosis, prognosis and treatment choices. The development and validation of valuable biomarkers can significantly improve the specificity, accuracy and efficacy of treatment for the HCC [18] Recent advancements in biomarker discovery for HCC hugely evolved in genomic, proteomic and immunological approaches to HCC biomarker identification thus transforming the management of the therapy [18]. The identification of biomarkers for HCC has progressed beyond scholarly and academia and gives essential clinical implications that could revolutionize patient care [18]. These biomarkers are necessary for tailoring personalised treatment and enhance the overall prognosis and outcomes. A study by Liebman et al. mentioned that serum DCP was present in patients with liver cancer in 1984, and since been shown over 90% of patients with liver cancer have high positive expression [19].

### Mechanisms of DCP production

Des-γ-carboxy prothrombin (DCP) is a prothrombin precursor developed in HCC, mainly due to its unique production mechanism in liver cell cancer. In HCC, due to lack of Vitamin K or γ-glutamyl carboxylase leads to incomplete carboxylation process of the 10 glutamic acid (Glu) residues in prothrombin precursor to γ-carboxylated glutamic acid (Gla) residues. As a result, some Glu residues remain uncarboxylated in the N-terminal domain, forming DCPs [20]. This abnormal variant of prothrombin lacks adequate and effective coagulation function and has been discovered to be potential autologous growth factor which can promote tumor growth mechanisms including DCP-Met-JAK1-STAT3 and foster invasion and metastasis through matrix metalloproteinases (MMPs) and ERK1/2 MAPK signaling pathways [19, 20].

### Role of DCP in HCC growth and invasion

Through the DCP-Met-JAK1-STAT3 signaling pathway, DCP induces formation of HCC. DCP promotes DNA synthesis in Hep3B and SK-Hep-1-cells by attachment of DCP to the c-Met-receptor at Tyr 1234/1235 which trigger autophosphorylation and stimulates downstream signaling [21]. This binding also promotes transcriptional activity by inducing STAT3 activation. DCP-induced transcription and cell growth can be prevented by blocking STAT3 with siRNA or targeted peptides. These results highlight the essential role of the DCP-Met-JAK1-STAT3 major signaling pathway in promoting progression of HCC, underlining the potential as a therapeutic target [20–22].

Metastasis and invasion, specifically intrahepatic spread, are main contributors to the poor prognosis of HCC. Increased invasion and metastasis in HCC, hepatic vein tumor thrombosis and invasion tumor in portal vein are likely to elevate DCP levels [23]. Studies have shown that DCP causes the HCC cells to release MMPs [24]. In the experiment with SK-Hep cells, exposure to DCP particularly raises their migration and invasion through Matrigel, suggesting active MMP breakdown. Western blot and Gelatin zymography analyses of cell supernatants verified that DCP caused the release of MMPs, specifically MMP-2 and MMP-9. With these findings, it highlights that DCP plays a crucial role in HCC growth, invasion and metastasis by modulating MMP activity [20, 24].

### Roles of DCP in angiogenesis of HCC

**Fig. 1:**
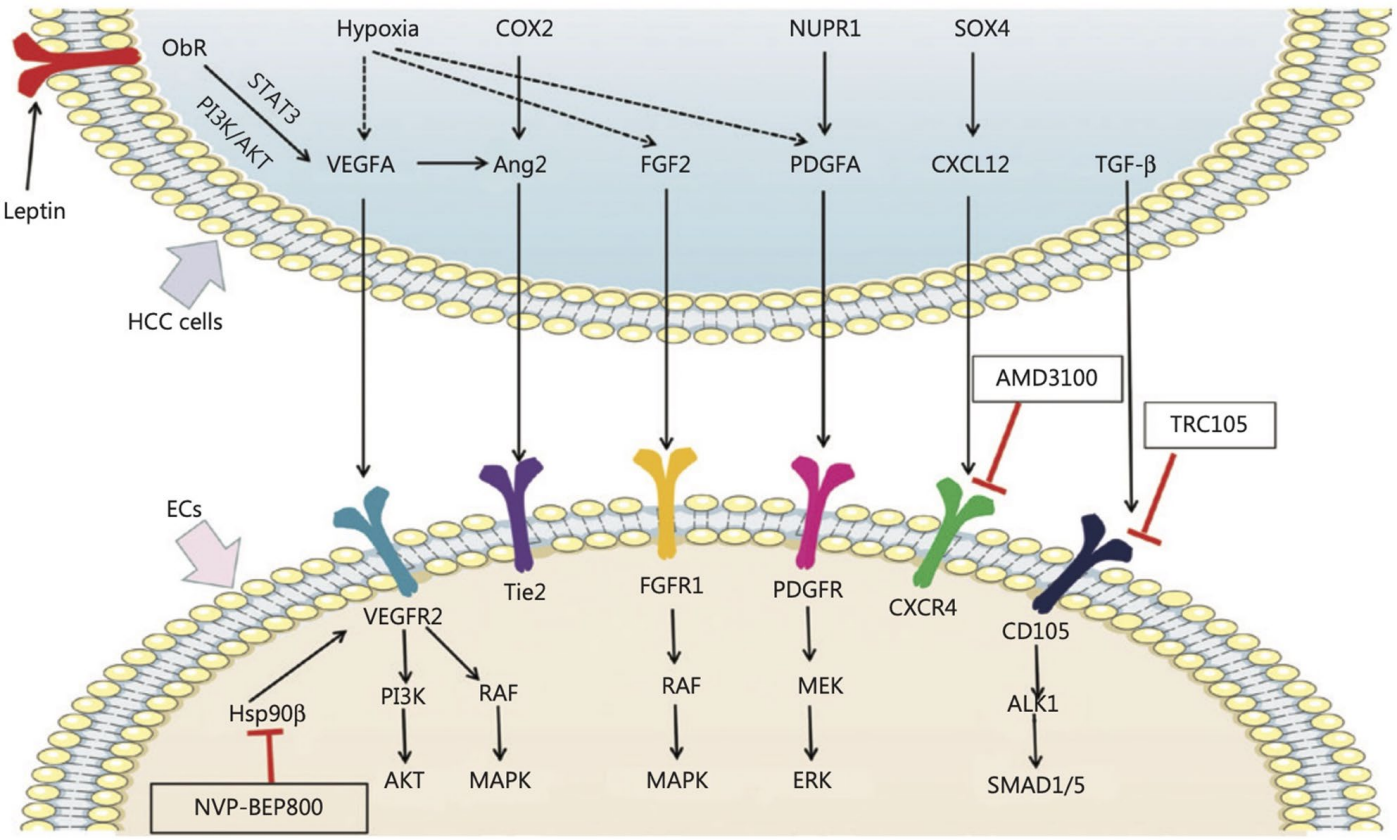
Mechanisms of pro-angiogenic factors inducing angiogenesis and new potential targets inhibiting angiogenesis in HCC. Proangiogenic factors secreted by HCC cells, including VEGFA, Ang2, FGF2, PDGFA, CXCL12, and TGF-β, bind the receptors expressed on ECs and promote angiogenesis in HCC. Hypoxia promotes expression of VEGFA, FGF2, and PDGFA in HCC cells. Leptin also promotes VEGFA expression by binding Ob-R in HCC cells. VEGFA binds VEGFR2 and subsequently activates the PI3K/AKT and RAF/MAPK pathways, thereby promoting angiogenesis. By interacting with FGFR1, FGF2 promotes angiogenesis in HCC by activating the RAF/MAPK pathway. PDGFA activates MEK/ERK signaling via PDGFR, thus promoting angiogenesis. TGF-β binds CD105 and activates the ALK/SMAD1/5 pathway, which promotes angiogenesis. COX2 and VEGFA induced Ang2 promotes angiogenesis by binding the Tie2 receptor. SOX4 induced CXCL12 promotes angiogenesis by binding the CXCR4 receptor. Nvp-bep800 inhibits HSP90β in ECs and subsequently attenuates angiogenesis in HCC. AMD3100 inhibits CXCR4 on ECs, thus attenuating angiogenesis in HCC. TRC105 inhibits CD105 on ECs and consequently attenuates angiogenesis in HCC. Reprinted from [29] under the terms of the Creative Commons Attribution License (CC BY) 4.0

HCC is predominantly a hypervascular tumor, with angiogenesis strongly linked to its invasion and metastasis progression [25]. Angiogenesis is crucial to HCC tissues for their sustained growth and deteriorates tumor behaviour and becomes more aggressive. Angiogenesis begins when cancerous cells start to release angiogenic factors produced by cancer cells, infiltrating cells and vascular endothelial cells [26]. In stromal space, these factors may stimulate further the migration and proliferation of vascular endothelial cells, resulting in the solid endothelial cells sprout formation [26]. Nowadays, there are numerous molecules that have been discovered in HCC-related angiogenesis including endothelial growth factor receptor (EGFR), vascular endothelial growth factor (VEGF), fibroblast growth factors (FGFs), transforming growth factor b (TGF-b), Fibroblast growth factors (FGF) and matrix metalloproteinases (MMPs) etc. Recent researches propose that DCP may interact with HCC cells or vascular endothelial cells and influencing the secretion and release of angiogenesis-linked molecules may serve as novel angiogenic factor, making it serve as a novel angiogenic factor (Fig. 1) [27–29].

### Application of personalised medicine in HCC based on pathophysiology

The application of personalized medicine in HCC can greatly benefit and advance by understanding the roles of DCP in promoting tumor growth and metastasis by serving as both a diagnostic and prognostic biomarker. Increased DCP is closely linked with advanced stages of HCC, indicating it has a pivotal role in early detection and evaluation of the risk. Integrating DCP measurement into clinical settings allows healthcare professionals to identify patients who might need more intensive and comprehensive treatment or more frequent follow-up. For example, it has been demonstrated that DCP diagnostic threshold of 60 mAU/ml improved specificity and sensitivity, allowing patient-individualized interventions [30].

Other than its diagnostic role usefulness, DCP offers important guidance in therapeutic decision making. Its function is biologically in tumor advancement and metastasis through specific pathways such as DCP-Met-JAK1-STAT3 signaling pathway [20]. By understanding this function, it will highlight the possible application of targeted therapy on DCP biomarkers that can block these mechanisms within strategies of personalised treatment. Moreover, combining other biomarkers such as AFP and GPC3 with DCP contributes to advanced and strong diagnostic panels designed to the specific molecular properties of an individual’s tumor [31, 32].

Next, HCC management is developed further by innovative tools such as GALAD score, which incorporates clinical parameters with biomarker levels such as DCP, AFP and GPC3 [33]. These stratification algorithms according to patients’ likelihood of developing HCC, thus allowing timely and personalized therapy [33, 34]. Additionally, another layer of accuracy is further improved the personalised medicines by the advancement in liquid biopsy technology, which include the incorporation of analysis of serum biomarkers and circulating tumor DNA (ctDNA)-use in early diagnosis of lung cancer and response monitoring [35, 36].

By utilising these cutting-edge diagnostic approaches, healthcare professionals and clinicians can tailor treatment to the unique biological characteristics of each patient’s cancer, significantly resulting in the outcomes improvement in HCC care.

### Diagnostic performance and efficacy with AFP

The diagnostic performance of DCP has been thoroughly verified in clinical studies, which has demonstrated superior sensitivity and specificity compared to AFP in diagnosis of HCC. Specifically, DCP shows an 87% sensitivity and 92% specificity, particularly in instances linked to chronic liver disease [37]. This enhanced performance is linked to the ability of DCP to detect or identify tumors whereas AFP is not able to identify effectively due to low or undetectable AFP levels [37]. Additionally, DCP proves its advantages with higher positive predictive value in diagnosing HCC cases with chronic Hepatitis B and C [38].

Moreover, there are longitudinal studies that also underline the usefulness of DCP beyond the first diagnosis. Repeated measurement of DCP has been shown to be effective in forecasting disease progression and recurrence following treatment, establishing it as an advantageous tool for monitoring HCC patients who are receiving liver transplantation or resection [31].

When compared to AFP, a well-established and longstanding biomarker for HCC diagnosis, DCP demonstrated its superiority, particularly in identifying early-stage HCC and differentiating malignant tumors from benign liver characteristics. Based on a multi-center study indicated that DCP was elevated in 91% of HCC cases while the AFP rose only 61% [38]. This percentage comparison clearly shows that DCP could be more efficient in recognizing a broader range of tumor phenotypes and underscores the needs of DCP in clinical settings to improve the diagnostic purpose of HCC [37]. However, there are studies that mentioned that diagnostic accuracy and patient care can be improved by combining DCP with other biomarkers [31, 32, 39].

## Glypican-3 (Gpc3)

In personalised medicine, identifying targetable tumor-specific or tumor-associated features to broaden therapy options in cancer patients is immensely useful in increasing the patient’s survival rate by early detection of the cancerous cell. Other than for diagnostic purposes, there are also biomarkers that are helpful for both diagnostic and therapeutics (theranostics). One of the developing cancer biomarkers that is being studied for its theranostic purpose in hepatocellular cancer is GPC3. GPC3 is a protein that is usually found in the placenta and the liver during fetal development but is rarely found in healthy adult tissues [40]. Eventually it is found that in patients who were diagnosed with HCC, this protein resurfaces and can even be detected in the blood. GPC3’s presence in liver cancer but absence in normal liver tissue enables it to distinguish between malignant and non-cancerous liver modifications. According to studies by [41], GPC3 is overexpressed in 77% of HCC cases, and a soluble form (sGPC-3) may be detected in the blood, allowing for noninvasive identification. The cut-off values for glypican-3 was two, i.e. 2.5 ng/mL to exclude HCC with an optimal sensitivity of 85% and 33.7 ng/mL for confirmation of HCC, with a specificity of 96.7% [42]. Thus, making it a promising biomarker that is useful in diagnosing this cancer.

### Glypican-3 in diagnosis of HCC

There are many other surface molecules that is elevated in HCC including transferrin receptor (TfR), asialoglycoprotein receptor (ASGP-R), AF20 antigen, somatostatin receptor (SSTR), and lysosome-associated protein transmembrane 4β (LAPTM4B). Nevertheless, it appears that their use in the diagnosis of HCC is restricted. GPC3, on the other hand, seems like a potential option because of its distinct qualities. GPC3 is not found in diseased liver cells including cirrhotic cells, hepatitis, and fatty liver disease, and it is also rarely expressed in adults [43]. In a study by Chen et al.,(2013), it was revealed that among 1037 subjects that included HCC patients, liver cirrhosis patients, non-HCC cancer and healthy people, the average serum GPC3 in HCC patients was significantly higher than subjects with other conditions [18].

### Glypican-3 role in solid tumor recognition and therapy

CAR T-cell therapy, which includes altering the immune system of a person to attack cancer cells, has been extremely efficient in treating blood cancers such as B cell malignancies. However, its success has been restricted in treating solid tumours due to a number of distinct obstacles such as lack of specific targets as the solid tumors often lack antigens that are both highly expressed and exclusive to tumor cells. This increases the risk of CAR T cells attacking healthy tissues, leading to off-target effects [10]. Recently, CAR macrophages (CAR-Ms) are being investigated by researchers as a potential substitute for CAR-T cells in the treatment of cancer, particularly solid tumours. According to the in-vivo and in-vitro studies, CAR-Ms, as opposed to CAR-T cells, capitalise on macrophages’ innate capacity to phagocytose tumour cells and enter tumours deeply. However, in CAR-M therapy, the immunosuppressive tumour microenvironment (TME) is the main barrier because it has the ability to rewire macrophages to adopt the M2 phenotype, which promotes tumour growth, rather than the M1 phenotype, which combats tumours [44].

**Fig. 2:**
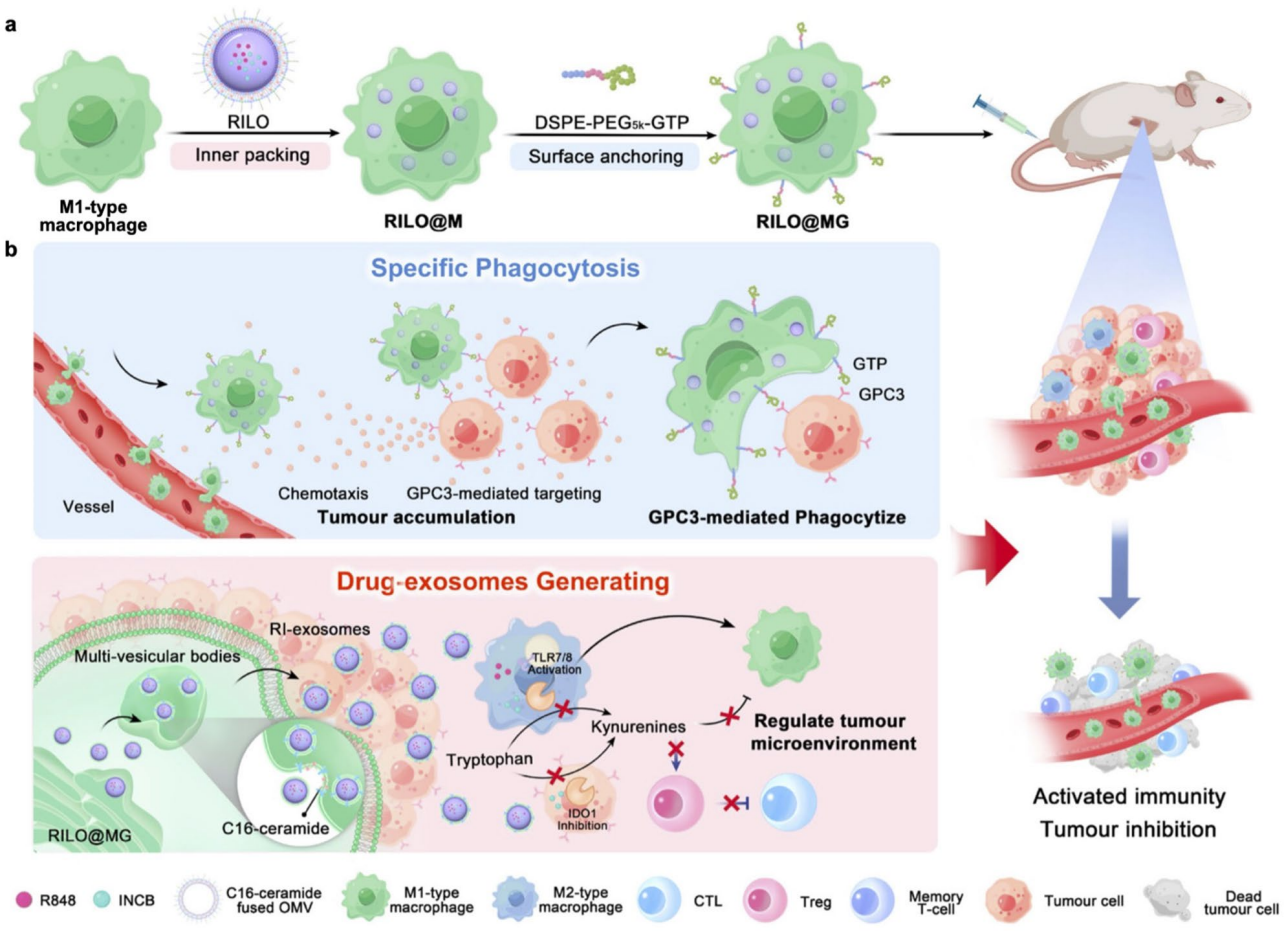
Schematic illustration of RILO@MG promoting specific tumour phagocytosis and generating drug exosomes that play a role in triggering an antitumour immune response to combat solid tumour.** a** Preparation of RILO@MG. First, RILO@M was prepared by the inner packing of M1-type macrophages and RILO. Then, DSPE-PEG5k-GTP was anchored on the surface of RILO@M to prepare RILO@MG. **b** RILO@MG accumulated in the tumour site through chemotaxis and GPC3-mediated targeting after i.v. administration directly killed tumour cells by GPC3-mediated phagocytosis and generated RI-exosomes con- taining R848 and INCB to regulate the TAM phenotype and enhance T-cell viability. Therefore, RILO@MG exerted antitumour efficacy by directly killing tumour cells and reversing the suppressive TME. CTL cytotoxic T-cell. Reprinted from [19] under the terms of the Creative Commons Attribution License (CC BY) 4.0

As a result, researchers intend to create M1 macrophages with more potent anti-tumor activity in order to increase the effectiveness of macrophages since they have the ability to attack tumour cells directly, release cytokines, which are substances that enhance immunity, and activate the immune system as a whole. However, M1 macrophages frequently lack selectivity, they may target both tumour and healthy cells. To increase their efficacy, researchers are working to improve tumor-specific targeting. As GPC3 is only expressed on the surface of the cancer cells (as in the HCC), it creates a huge potential in advancing this kind of therapy. By affixing GPC3-targeting peptides to macrophages, scientists hope to improve these immune cells’ capacity to identify and bind to tumour cells that produce GPC3 [19] (Fig. 2). They developed a macrophage-based therapeutic system engineered to target and combat solid tumors by carrying surface GPC3-targeting peptides (GTP) and encapsulated R848/INCB024360-loaded lipid OMVs (RILO) for enhanced tumor specificity and therapeutic delivery. This strategy aims to improve the likelihood that macrophages will recognise and target cancer cells while avoiding healthy tissues and to enhance the phagocytosis of tumours, which engulfs and eliminates cancer cells. By concentrating on tumours with elevated GPC3 expression, cancer treatments can be made more effective overall.

### Glypican-3 tumor vaccines

Other than that, GPC3 can also be developed into a tumor vaccine. In the development of vaccines, usually it requires modifying a patient’s dendritic cells in order to recognize tumor-specific antigen (TSA) before introducing it back to the patient’s body [43]. One of the developing vaccines for HCC is peptide-based vaccines. Several preclinical and clinical studies have examined peptide-based vaccines aimed at various HCC-related antigens, with glypican-3 (GPC3) being a prominent focus. In a phase I clinical trial of 33 advanced HCC patients, the GPC3 vaccination was well tolerated and successfully generated GPC3-specific immune responses in the vast majority of participants. These trials show that GPC3 has a potential to be developed as a vaccine and might be useful for the treatment of hepatocellular carcinoma.

## Challenges

### Issues on the usage of biopsy methods for HCC

**Fig. 3:**
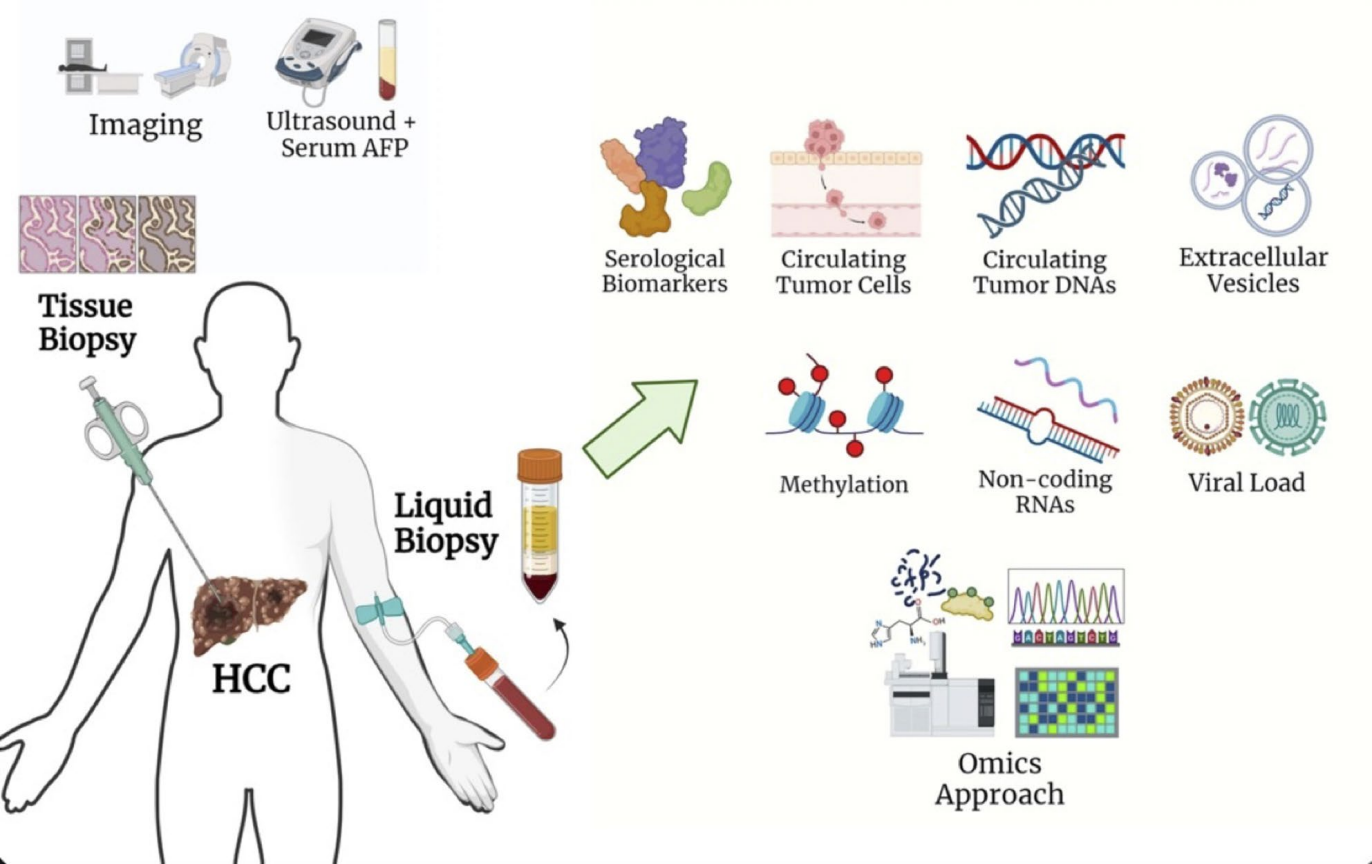
The biomarkers of hepatocellular carcinoma diagnosis. The current surveillance methodology for Hepatocellular Carcinoma (HCC) primarily involves ultrasound and serum AFP measurements, sometimes supplemented with imaging techniques. In instances where diagnosis or treatment options are ambiguous, tissue biopsy is often employed to confirm the histological structure of the tumor. Liquid biopsy offers a minimally invasive alternative for gaining insights into tumor heterogeneity. Recent research has identified potential biomarkers from diverse sources, including serological components, genetic materials, cells, and vesicles. Among these candidate biomarkers, circulating tumor DNAs and omics stand out due to their ability to reflect the complexity of HCC tumors. These technologies offer great promise in understanding the intricacies of HCC and developing more effective and personalized treatment strategies. The figure is reprinted from [45] under the terms of the Creative Commons Attribution License (CC BY) 4.0

First challenges that should be addressed regarding the usage of biomarkers in personalised medicine for HCC are issues and limitations found in each method for HCC diagnostics. There are a few methods for obtaining serum biomarkers to diagnose HCC. The typical main approach for HCC generally includes ultrasound imaging alongside serum AFP levels and, in certain situations, advanced imaging methods are required. If there is uncertainty regarding the diagnosis or treatment choices, a tissue biopsy could be performed to verify the histological features of the tumor to be more precise. Nonetheless, this method is lengthy as it requires several processes, and could be intrusive for the patient and make them uncomfortable [45] (Fig. 3).

An alternative method that could be used to avoid these problems is liquid biopsy, which is becoming more significant for cancer detection and tracking treatment outcomes. This approach is very beneficial as it offers real-time data with limited intrusiveness. Blood is the fluid most frequently used for liquid biopsy in clinical settings, however, there are also other bodily fluids used like urine, ascites, bile, and saliva [45]. Liquid biopsy also provides a broader range of data collection from the patient’s body. As shown in Fig. 1, this method can detect serological biomarkers, circulating tumor cells, circulating tumor DNA, extracellular vesicles, methylation patterns, non-coding RNAs, viral loads, and information obtained from omics strategies. Among these potential biomarkers, circulating tumor DNAs and omics are notable for their capacity to represent the complexity of HCC tumors.

However, there are certain issues that also need to be addressed regarding liquid biopsy methods. Initially, circulating tumor cells (CTCs) have been assessed as possible markers of treatment response in different types of cancer. Nonetheless, identifying CTCs is especially difficult in the initial phases of the disease. Secondly, circulating tumor DNA (ctDNA) represents another type of liquid biopsy; however, in typical circumstances, it makes up less than 1% of the overall circulating cell-free DNA. Moreover, ctDNA might not consistently indicate tumor-specific DNA; rather, it could derive from necrotic or apoptotic cells in the tumor or even from non-tumorous tissues. Thus, in these instances, recognising genetic or epigenetic alterations in DNA that are specific to cancer is essential. Moreover, concerning cirrhosis, these mutations might not be specific to HCC, since other DNA mutations or bacterial DNA can also be present in peripheral blood circulation [46].

Therefore, obtaining biomarker serum data to be used in personalized medications for HCC from a patient’s body using today’s techniques would pose considerable difficulties. Every method used to extract biomarkers has its own limitations, it either would be time consuming and intrusive for the patient or lacking accuracy and precision, which lead to potentially misleading information.

### Funding risk in research for personalised medicine

The second challenge found and need to be addressed is the risk of funding required to research biomarkers to develop personalised medicine for HCC. Just by detecting the biomarkers is not sufficient enough to proceed for the personalised medication for the patient and more steps need to be taken after. For instance, there is still the need for research after successfully detecting the presence of certain biomarkers for personalised medicine to understand the mechanism of the biomarkers found and to develop new treatments specifically for each different individual. One of the major challenges in establishing personalised medicine by using biomarkers for diagnostics is the need for significant funding to support research that is based on vast genomic data from the society. The availability of the infrastructure required to continue the experiment and to interpret genetic information for the development of personalised medicine is necessary [47]. Personalised medicine, which tailors disease prevention, detection, and treatment to an individual’s genetic information, necessitates large amounts of data from other patients’ genomes. This data helps researchers to better understand genetic variations and design personalised medicines according to the biomarkers found, but obtaining and managing it demands advanced infrastructure for both experimentation and interpretation. Given the sheer size of the human genome, which contains over six billion data points, managing and analysing this volume of information is difficult. Studies have shown that clinicians frequently find it challenging to understand such large data sets [47].

An example of the necessary infrastructure is the Genomic Data Infrastructure (GDI) project, launched in November 2022 across Europe, which was designed to overcome these challenges by developing a comprehensive genomic framework. This GDI project comprises three pillars, Pillar I focuses on long-term sustainability, Pillar II on delivering the critical 1 + MG infrastructure, and Pillar III which creates use cases to stimulate application and innovation. Through these three pillars, the GDI project promotes research and innovation, incorporates discoveries into clinical and healthcare practices, and advances public health efforts [48]. Without such infrastructure, the efficacy of genomic diagnostics and personalised therapy would be severely limited and restricted.

At the same time, ensuring thorough security measures are needed to protect the patients’ information, which requires a large amount of funding for advanced data protection technologies. Protecting the huge amount of sensitive genetic data acquired in personalised medicine is important for preventing data breaches and maintaining patient anonymity. Implementing a secure infrastructure capable of handling and protecting this volume of sensitive data requires ongoing investment of high-quality cybersecurity solutions. Additional money is also required to educate and train doctors in the proper use of these technologies, as well as to provide guidance on ethical practices to avoid potential problems.

Funding is especially critical in countries that lack the appropriate equipment to grow tissue cultures on their own, resulting in a reliance on imported cells from overseas. For example, Capricorn Scientific GmbH, an ISO-certified company based in Germany, manufactures cell culture products such as media, reagents, and sera for the use in biotechnology, diagnostics, and research [49]. However, when importing cell cultures, additional fees such as taxes, shipping, and currency conversion will further increase the overall cost. As a result, adequate financing is essential to cover these expenditures while also supporting the infrastructure required for tissue culture in personalised medicine.

## Future direction

Many emerging biomarkers are still in the early stages of research. Large-scale, prospective studies are needed to validate their clinical utility and establish standardized protocols [50]. The future trends in determining the biomarker of HCC is using machine learning and artificial intelligence (AI). It will be gaining significant traction of the biomarker. The trend is evident from the increasing number of studies utilizing these technologies to identify novel biomarkers and improve diagnostic accuracy [51].

## Conclusion

In conclusion, the growing body of research on utilizing biomarkers for HCC diagnostics and therapeutics has identified AFP, DCP, and GPC3 as some of the most promising candidates. These biomarkers have the potential to advance personalised medicine approaches, enabling more precise diagnostic and therapeutic strategies for HCC. However, the full scope of their capabilities still needs to be thoroughly studied before integrating these tumor biomarkers into standard HCC management protocols. For example, AFP, although already in use as an HCC biomarker, still has limitations in accuracy. However, combining AFP with PIVKA-II has been shown to significantly improve diagnostic precision, highlighting the potential of multi-biomarker approaches. Another critical consideration is the limitations and challenges associated with implementing these biomarkers, such as financial constraints and technical barriers. Therefore, the continuous development of advanced strategies is crucial to support the application of personalised medicine for the diagnosis and treatment of HCC using serum biomarkers. This will significantly enhance the understanding and management of HCC by providing more targeted diagnostic, therapeutic, and prognostic insights.

## Data Availability

No datasets were generated or analysed during the current study.
